# Dissecting the mechanisms and molecules underlying the potential carcinogenicity of red and processed meat in colorectal cancer (CRC): an overview on the current state of knowledge

**DOI:** 10.1186/s13027-018-0174-9

**Published:** 2018-01-15

**Authors:** Marco Cascella, Sabrina Bimonte, Antonio Barbieri, Vitale Del Vecchio, Domenico Caliendo, Vincenzo Schiavone, Roberta Fusco, Vincenza Granata, Claudio Arra, Arturo Cuomo

**Affiliations:** 1 Division of Anesthesia and Pain Medicine, Istituto Nazionale Tumori - IRCCS – “Fondazione G. Pascale”, Via Mariano Semmola, 80131 Naples, Italy; 2 S.S.D. Sperimentazione Animale, Istituto Nazionale Tumori - IRCCS - Fondazione “G. Pascale”, Via Mariano Semmola, 80131 Naples, Italy; 3 Division of Anesthesia and Intensive Care, Hospital “Pineta Grande”, Castel Volturno, Caserta, Italy; 4 Division of Radiology, “Istituto Nazionale Tumori - IRCCS - Fondazione G. Pascale”, Via Mariano Semmola, 80131 Naples, Italy

**Keywords:** Carcinogenesis, Red meat, Processed meat, Heme, Heterocyclic amines, Polycyclic aromatic hydrocarbons, Neu5Gc

## Abstract

Meat is a crucial nutrient for human health since it represents a giant supply of proteins, minerals, and vitamins. On the opposite hand, the intake of red and processed meat is taken into account dangerous due to its potential of carcinogenesis and cancer risk improvement, particularly for colorectal cancer (CRC), although it has been reported that also the contaminations of beef infected by oncogenic bovine viruses could increase colorectal cancer’s risk. Regarding the mechanisms underlying the potential carcinogenicity of red and processed meat, different hypotheses have been proposed. A suggested mechanism describes the potential role of the heterocyclic amines (HACs) and polycyclic aromatic hydrocarbons (PHAs) in carcinogenesis induced by DNA mutation. Another hypothesis states that heme, through the lipid peroxidation process and therefore the formation of N-nitroso compounds (NOCs), produces cytotoxic and genotoxic aldehydes, resulting in carcinogenesis. Furthermore, a recent proposed hypothesis, is based on the combined actions between the N-Glycolylneuraminic acid (Neu5Gc) and genotoxic compounds. The purpose of this narrative review is to shed a light on the mechanisms underlying the potential carcinogenicity of red and processed meat, by summarizing the data reported in literature on this topic.

## Background

**Fig. 1:**
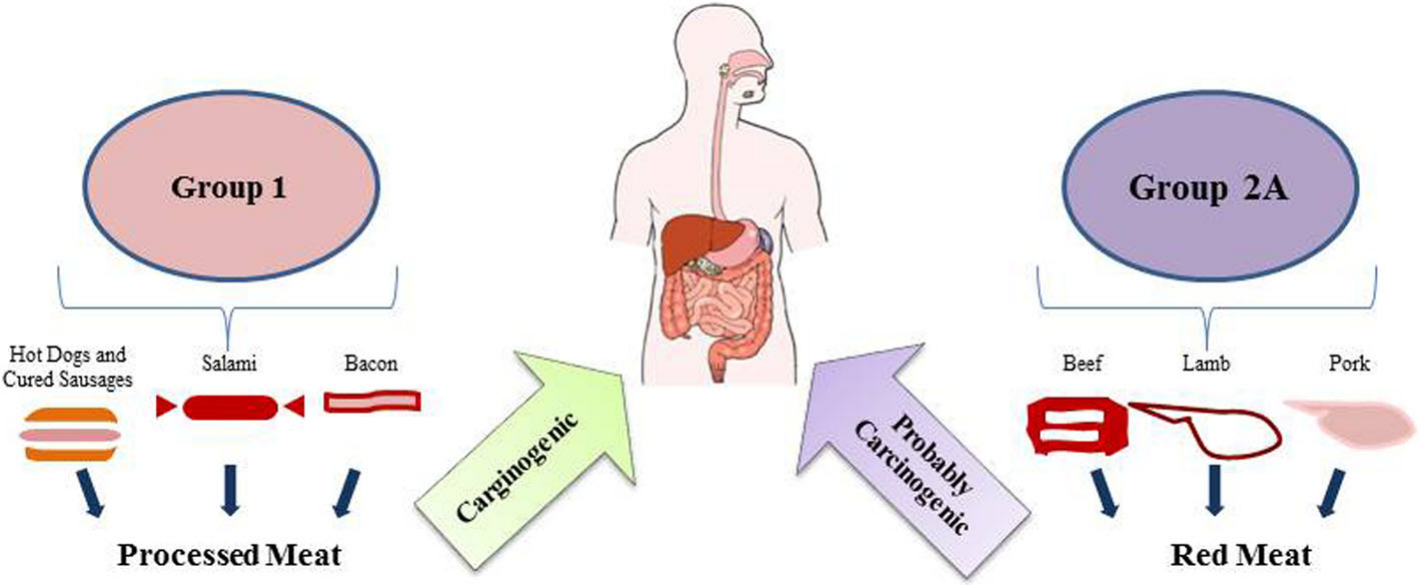
IARC’s classification of red and processed meat. Processed meat has been classified as carcinogenic to human (Group 1), while red meat has been classified as probably carcinogenic to human (Group 2A)

Meat is an important nutrient for human health, since it represents a big source of proteins, minerals and vitamins with poor bioavailability. Red and processed meats, instead, are considered dangerous, due to their potential carcinogenicity. Red meat is a form of unprocessed mammalians muscle, which color is due to the presence of myoglobin [1, 2]. On the other facet, processed meat is identified as a product obtained through several processes such as salting, curing, fermentation or smoking, with the aim of enhancing flavor or improve preservation. Recently, the International Agency for Research on Cancer (IARC) published interesting results on the potential carcinogenicity effects of red and/or processed meat; *Bouvard* et al. [3] anticipated these data and showed that the processed meat is classified as carcinogenic to human (Group 1), while the red meat is identified as probably carcinogenic to human (Group 2A) (Fig. 1**)**. On the basis of this classification, and from knowledge emerged by epidemiological studies, the intake of meat as a nutrient of a healthy diet for human, has to become a controversial issue [4]. Accumulating studies showed that the consumption of processed meat causes colorectal cancer (CRC), the second most common cause of cancer-related death in affluent countries, and stomach cancer, although the evidences for this latter are still not enthusiastic. *Chan* et al. [5] proved evidence that dietary factors, as well as red and processed meat, may be thought of the leading causes of 80% CRC cases. Similar results, obtained by epidemiological studies accumulated over the last decades, confirmed the association of red and processed meat intake and increased risk of CRC [6–9]. Interestingly, *Zu Hausen* et al., suggested that potentially oncogenic thermoresistant bovine viruses, by inducing beef’s contaminations provoked infections in the colorectal tract, that combined to chemical carcinogens developed during procedures of cooking, increased the colorectal cancer’s risk [10]. The role of red meat consumption as a leading cause of different types of cancer (e.g. pancreatic cancer, prostate cancer, bladder cancer) has been conjointly reported [11–16], although extremely mentioned and contested by the scientific community, since a recent meta-analysis indicated a significantly increased risk (about 22%) of bladder cancer as a result of high processed meat (but not for red meat) consumption [17]. Additionally, in another meta-analysis, *Bylsma and Alexander* concluded that there was no association between red or processed meat intake and prostate cancer, although they found a weak positive summary estimate for processed meats [18]. A detailed summary of recent epidemiological studies on carcinogenicity of consumption of red and processed meat in different types of cancer, including colorectal cancer (CRC), has been recently reported by *Domingo* et al. [19] whereas *Alexander* et al. published a quantitative update on the epidemiological research on the topic [20]. Several molecules have been identified as potential carcinogenic present in red meat or produced by meat processing or by cooking procedure: i) the heterocyclic amines (HACs), as -Amino-3,4- dimethylimidazo quinolone (MeIQ) and 2-Amino-3,8-dimethylimidazo quinoxaline (MeIQx), ii) the N-nitroso-compounds (NOCs), as N-nitrosodimethylamine (NDMA); iii) the polycyclic aromatic hydrocarbons (PAHs), as benzo[a]pyrene (BaP) [17, 21]; iv) the N-glycolylneuraminic acid (Neu5Gc) [22]. In addition, the carcinogenic role of environmental pollutants (e.g., polychlorinated dibenzo-pdioxins and dibenzofurans, polychlorinated biphenyls, polybrominated diphenyl and polychlorinated diphenyl ethers, polychlorinated naphthalene and perfluoroalkyl substances), which are already present in raw or unprocessed meat, has been also proposed [19]. Furthermore, the carcinogenic role of red and processed meat could be enhanced by other concomitant dietary factors (e.g., high fat and/ or protein intake) and clinical conditions, such as obesity [23, 24]. It is of note that bile acids produced in the gut as a consequence of high fat intake, by damaging the mucosa and the epithelium of colon, leads to cell hyper-proliferation and then to colon cancer development [25]. Despite studies on animal models supporting the cancer-promoting effect induced by high fat intake [26], epidemiological studies reported opposite results [27, 28]. Similarly, a high protein intake provokes the formations of metabolites toxic with a great potential of colon carcinogenesis [29], although no data supporting this hypothesis, are available [30]. Others alternative and uncertain hypothesis of mechanisms (e.g., thermoresistant potentially oncogenic bovine viruses [10] and endogenous hormones, [31]) underlying the consumption of red and processed meat and carcinogenesis of CRC, have been reported in the literature and reviewed by *Demeyer* et al. [32]. Our aim is to review the data reported in the literature on the principal mechanisms and molecules involved in carcinogenicity induced by red and processed red meat consumption in CRC, in order to shed a light on the current state of the art on this important issue.

### Mechanisms and molecules involved in the carcinogenicity induced by consumption of red and processed meat

The biological reasons for the association between red and processed meat and cancer- especially CRC- are still unclear, but a large number of molecular mechanisms have been proposed to explain this association. Here, we summarize the most relevant ones, trying to elucidate the current state of scientific knowledge.

#### Heterocyclic amines (HCAs)

Heterocyclic amines (HCAs) represent chemical compounds generated in fish and meat by cooked procedures at high temperatures through a specific reaction (i.e. Maillard reaction [33]) between free amino acids and sugars, becoming in this way potentially mutagens to humans [34]. It has been reported that the principals HCAs found in cooked red meat (over a total of 25 identified) are the 2–Amino-3, 8-dimethyl imidazo-[4,5f] quinoxaline (MeIQx) and the 2-Amino-1-methyl6 phenylimidazo [4,5b] pyridine (PhIP) [35]. These compounds, together with amino-3,4- dimethylimidazo[4,5-f] quinoline (MeIQ), have been classified by the IARC as potential carcinogenic to human (Group 2B), while the amino-3-methylimidazo[4,5-f] quinolone (IQ) has been classified as probably carcinogenic to human (Group 2A) [3]. It is of note that HCAs are transformed into mutagens after metabolic activation which regulates their carcinogenicity [8]. Evidences support the hypothesis that the levels of these compounds are high in human organisms after the consumption of cooked red beef [36]. Thanks to in vitro and in vivo pre-clinical studies the metabolism and the molecular pathways of MelQx and PhIP’s biotransformation have been largely dissected [37–40]. Specifically, the process of hydroxylation of MelQx and PhIP mediated by the cytochrome P-450, has been identified as the principle pathway [41]. *Schut* et al. [42] showed that HCAs may form DNA adducts. Several epidemiological studies reported a strong association between the HCAs intake and CRC [43–45]. *Le Marchand* et al. confirmed these data as they reported a strong association between MeIQx and rectal cancer [46]. Unfortunately, opposite results have been described probably due to the higher variability of other factors responsible for carcinogenesis (e.g. diet, genetic polymorphisms of the population) used in epidemiological studies [47, 48]. On the basis of these contrasting results, the link between HCAs and cancer risk is not completely clarified [34]. Thus, more studies will be needed.

#### The polycyclic aromatic hydrocarbons (PAHs)

Polycyclic aromatic hydrocarbons **(**PAHs) are considered toxic substances produced by an incomplete combustion of organic compounds such as tobacco, oil, gas. [49]. Regarding the red meat and other foods, PAHs are produced by cooking procedures at high temperatures, such as barbecuing, or by the food’s processing by using smoking [50]. The principal PAH (over 100 identified) classified by the IARC as potential carcinogenic to human (Group 1) is the benzo[a]pyrene (BaP) [3]. *Estensen* et al., in a pre-clinical study conducted on mice with lung tumors, confirmed the carcinogenic role of BaP [51]. This molecule becomes genotoxic after metabolic reaction by which is converted into benzo[a]pyrene diol-epoxide (BPDE) [52]. This latter is able to interfere with the bases of DNA, thus resulting in DNA damage which is responsible for cancer promoting [53, 54]. Regarding the carcinogenic mechanism of PAHs, *Phillips* et al.*,* showed that human colon cells are able to metabolize these compounds [50]. *Shimada* et al. demonstrated that the aryl hydrocarbon receptor, a transcription factor activated by ligands like PAH, plays a crucial role within the regulation and therefore the drug metabolizing enzymes (e.g. CYP1A1, CYP1A2, CYP1B1, glutathione S-transferase and UDP-glucoronyltransferase). These enzymes cause toxicity or carcinogenesis through the processing of the toxicants to reactive metabolites that, finally, interact with cellular macromolecules (e.g., DNA adducts) [55]. Similarly, to HCAs, epidemiological studies do not rumor convincing results on the association between dietary BaP intake from meat and cancer appearing (mainly CRC), most likely as a result of the difference in un-supporting factors [56, 57] (e.g. cooking procedures, fat contents) chosen in the populations listed in these studies.

Thus, as for HCAs, additional studies are going to be necessary to ascertain the carcinogenicity of red and processed meat induced by PAHs.

#### Heme

**Fig. 2:**
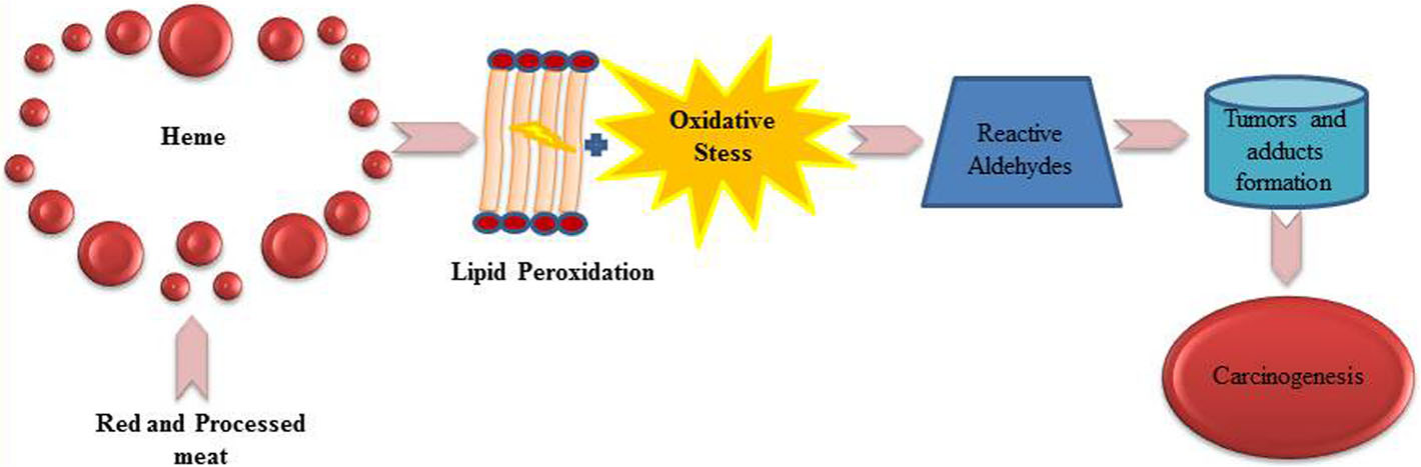
Lipid peroxidation as a mechanism underlying the carcinogenicity of red and processed meat induced by heme. Heme induces lipid peroxidation trough oxidative stress, resulting in the formations of reactive aldehydes. These cytotoxic aldehydes, cause carcinogenesis by promoting the tumors and therefore the adducts formation

Heme represents the prosthetic cluster of myoglobin and hemoglobin [28] and is responsible for the red color of meat, as a results of its elevated concentrations respect to those observed in white meat [58]. Due to many epidemiological [59–61] and pre-clinical studies [62, 63], three mechanisms underlying the association between a consumption of heme and CRC risk, have been elucidated: i) the lipid –peroxidation; ii) the *N*-nitroso compounds (NOCs) formations; iii) the cytotoxicity. The potential carcinogenicity of heme iron may be associated to its redox properties. By taking part in dangerous free radical-generating reactions with the production of a reactive oxygen species (ROS), heme iron leads to oxidative DNA damage which is considered highly mutagenic [64]. ROS are involved in lipid peroxidation, a complex process which, finally, causes the formation of cytotoxic and genotoxic aldehydes, as malondialdehyde (MDA) and 4-hydroxynonenal (4-HNE) [65]. These aldehydes are able to promote cancer progression, as reported by epidemiological and experimental studies. Thus**,** lipid peroxidation as thought of one the principal mechanisms underlying the carcinogenicity of red and processed meat induced by heme (Fig. 2).

#### The nitrate/nitrite formation and the N-Nitroso compounds (NOCs)

**Fig. 3:**
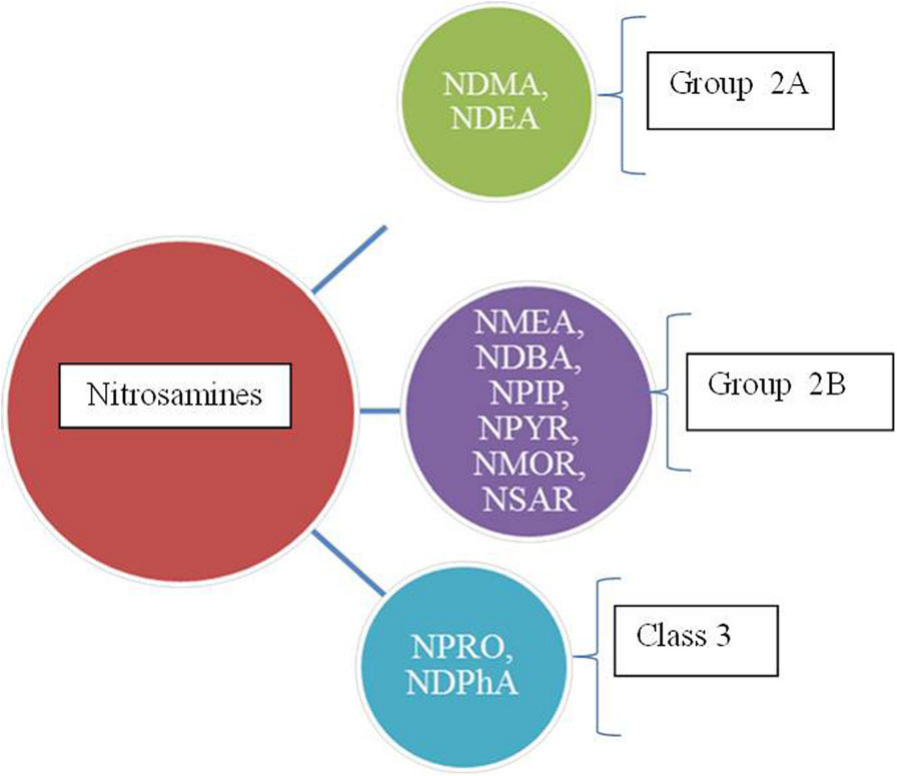
Classification of Nitrate/Nitrite and N-Nitroso compounds from IARC. NDMA, NDEA have been classified as probably carcinogenic to humans (Group 2A); NMEA, NDBA, PIP, NPYR, NMOR, NSAR have been classified as possibly carcinogenic to human; NPRO, NDPhA have been not classified as to its carcinogenicity to human (Group 3) [3]. Abbreviations: NDMA: N-nitrosodimethyamine; NDEA: N-nitrosodiethylamine; NDBA: Nnitrosodibutylamine; NPIP: N-nitrosopiperidine; NPYR: N-nitrosopyrrolidine; NMOR: Nnitrosomorpholine; NDPhA: Nnitrosodiphenylamine; NPRO: N-nitrosoproline; NSAR: Nnitrososarcosine. Group 2A: probably carcinogenic to human; Group 2B: possibly carcinogenic to human; Group 3: not classifiable as to its carcinogenicity to human

Endogenous NOCs, formed by N-nitrosation process of amines and amides, may be considered important genotoxins since being to induce DNA mutations [66]. Most NOCs, as well as nitrosamines, nitrosamides, and nitrosoguanidines [67], can yield alkylating agents (N-alkyl-NOCs) throughout metabolism. Plenty of nitrosamines are found in foods, including NDMA, NDEA (Nnitrosodiethylamine), NDBA (N-nitrosodibutylamine), NPIP (N-nitrosopiperidine), NPYR (Nnitrosopyrrolidine), NMOR (N-nitrosomorpholine), NDPhA (Nnitrosodiphenylamine), NPRO (Nnitrosoproline), and NSAR (N- nitrososarcosine), although not all of these shows carcinogenic effect [3] (See Fig. 3, for IARC’s classification of Nitrosamines). Humans may be exposed to two different forms of NOCs: i) exogenous, derived from different sources (e.g., tobacco products, diet, occupational environments and drugs); ii) endogenous nitrosamines and nitrosamides, generated by the reaction of nitrite with the products of amino acid’s degradation in the stomach, and accounted for up 75% of the total NOC exposure [68]. Several epidemiological and pre-clinical studies conducted on animal models reported a strong link between endogenous NOCs and CRC.

Regarding CRC, studies demonstrated that N-alkyl-NOCs can induce transitions of DNA’s bases (GC → AT) in genes mutated (e.g. *Kras*) in human tumors [69]. Moreover, as shown by *Kuhnle* et al. [70], the presence of nitrosyl heme, which is formed by a nitrosylation or nitrosation in ileum and in faeces, might promote the formation of extremely reactive alkylating agents like diazoacetate. As a consequence, this process ends up in the formation of the NOC DNA adduct, named O6-carboxymethyl-2′-deoxy-guanosine (O6CMeG). This compound has been wide studied by *Lewin* et al., as carcinogenetic agent for CRC [71]. DNA damage may be induced by aldehydes with mutagenic effects in microorganism, mammalian, and human cells [72]. For example, *Leuratti* et al. [73]*,* proved evidence that MDA reacts with DNA to form adducts such as 1, N2-malondialdehyde-deoxyguanosine (M1dG), which has been found at higher levels, in subjects with adenoma compared with adenoma-free subjects. On the other side, the aldehydes 4-hydroxy-2-nonenal (4-HNE) is weakly mutagenic even though is taken into account the most toxic product of lipid peroxidation. In fact, it may interfere with stress apoptosis pathway by causing necrosis in human colon carcinoma cells through the activation of caspase-3 [74]. To overcome the pro-carcinogenic effects of heme, it’s been powerfully steered to incorporate in dietary regime a spread of molecules present in fruit and vegetables. As an example, calcium salts and chlorophyll are able to precipitate heme molecules, whereas vitamins C and E blocked the endogenous formation of NOCs, and several other polyphenols like quercetin, α-tocopherol, or red wine polyphenols, suppressed the lipid peroxidation.

Fewer pre-clinical studies conducted on animal models, showed that heme is able to augment the citoxocity of colon cells, resulting in an increased epithelial proliferation and afterward to cancer occurring [10, 75]. Unfortunately, few epidemiological studies [63, 76] confirmed these knowledges resulting in interpretations that lack of consistency.

#### N-glycolylneuraminic acid (Neu5Gc)

Red meat is enriched in glycan’s containing a variant of sialic acid, the N-glycolylneuraminic acid (Neu5G). This molecule, not naturally found in human tissues, may be solely assumed by diet regimens containing pork, beef, and lamb [22, 77]. To the current issue, it has been suggested that humans can metabolically incorporate and express Neu5Gc into cell surface glyco-conjugates [78]. Moreover, it has been reported that the incorporation of Neu5Gc into human tissue could be involved in tumor initiation and progression [79]. The mechanism of uptake and incorporation of Neu5Gc into human epithelial was described by *Bardor* et al., 2005) [80]. It is necessary to underline that Neu5Gc-containing glycans act as “xeno-autoantigens” that may be targeted by naturally circulating anti-Neu5Gc “xeno-autoantibodies”. This process ends up in development of xenosialitis, an inflammatory disease that influences cancer formation and progression [81, 82].

Due to the absence of epidemiological data, is suitable to consider the xenosialitis induced by Neu5Gc assumed by red meat consumption, as the only mechanism underlying the association between red meat consumption and increased CRC risk.

## Conclusion

**Fig. 4:**
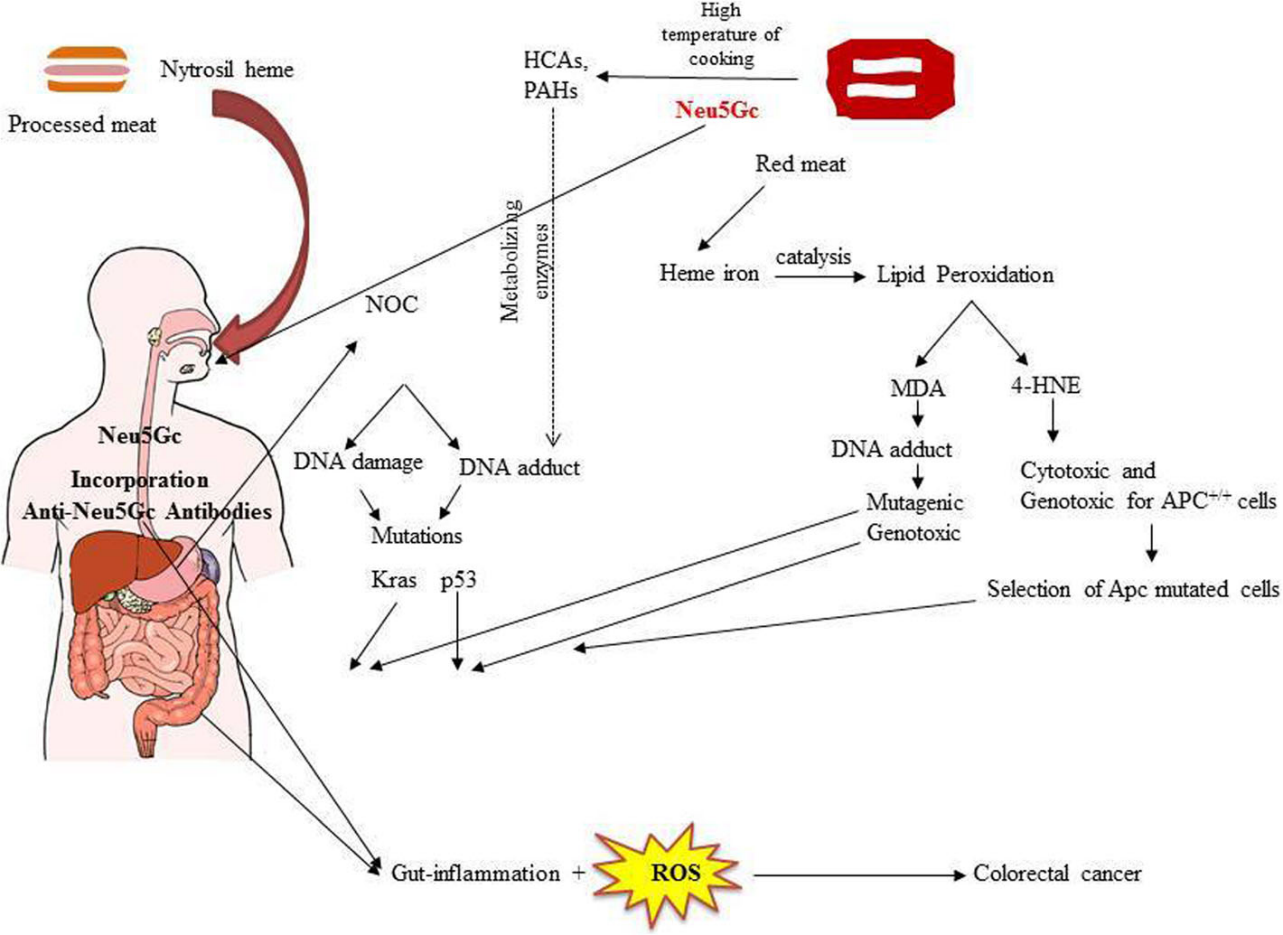
Mechanisms underlying the carcinogenesis of red and processed meat in CRC. Polycyclic aromatic hydrocarbons (PAHs) and heterocyclic amines (HCAs) produced after cooking procedures at hight temperatures, after metabolic activation, lead to the formation of DNA adduct in epthelial cells with great potential carncinogenesis to CRC. Heme, by lipid peroxidation and endogenous formation of N-nitroso compounds *(*NOCs), provokes several DNA mutations responsible for CRC. Then, an inflammation process generated by an association of products of lipid peroxidation induced by heme, reactive oxygen species (ROS) present in red and processed meat, and Neu5GS, could enhance the CRC development

In 2015 the IARC classified the consumption of red meat and processed meat as “probably carcinogenic to humans” (Group 2A), and as “carcinogenic to humans” (Group 1), respectively. A large number of mechanisms have been proposed to elucidate the link between red and processed intake and CRC risk. These mechanisms involve different molecules: i) heme iron, ii) NOCs, iii) HCAs and PAHs; iv) Neu5G, although convincing results have been reported for heme (via lipid peroxidation mechanism) and endogenous NOC. Taking into the account that different compounds could also be present in red and processed red meat, the increased risk for CRC could also be related to multiple carcinogenic compounds (Fig. 4). More studies are going to be necessary to elucidate the molecular mechanisms underlying the carcinogenicity of red meat and processed meat in CRC and other types of cancer.

## Abbreviations

(Neu5Gc): N-Glycolylneuraminic acid
(NOCs): N-nitroso compounds
4-HNE: 4-hydroxy-2-nonenal
Bap: Benzo[a]pyrene
CRC: Colorectal cancer
HCAs: Heterocyclic amines
IQ: Amino-3-methylimidazo[4,5 f]quinolone (IQ)
M1dg: 1,N2-malondialdehyde-deoxyguanosine
MeIQ: amino-3,4-dimethylimidazo[4,5-f] quinolone
MeIQx: 2–Amino-3,8-dimethylimidazo-[4,5f]quinoxaline
NDBA: N-nitrosodibutylamine
NDEA: Nnitrosodiethylamine
NDMA: N-nitrosodimethyamine
NDPhA: N-nitrosodiphenylamine
NMOR: N-nitrosomorpholine
NPIP: N-nitrosopiperidine
NPRO: N-nitrosoproline
NPYR: N-nitrosopyrrolidine
NSAR: N-nitrososarcosine
O6CMeG: O6-carboxymethyl-2′-deoxy-guanosine
PHAs: Polycyclic aromatic hydrocarbons
PhIP: 2-Amino-1-methyl6 phenylimidazo[4,5b]pyridine
ROS: Reactive oxygen species
